# Translation and psychometric validation of the Arabic version of Summary of the Diabetes Self-Care Activities (SDSCA) among pregnant women with gestational diabetes

**DOI:** 10.1186/s12884-022-04897-4

**Published:** 2022-07-14

**Authors:** I. Al Hashmi, H. Al-Noumani, F. Alaloul, S. Murthi, A. Khalaf

**Affiliations:** 1 College of Nursing, Sultan Qaboos University, Muscat, Oman; 2 Oman Medical Speciality Board, Muscat, Oman; 3 Faculty of Health Sciences, Kristianstad University, Elmetorpsvägen 15, SE-291 88 Kristianstad, Sweden

**Keywords:** Validation, Instrument, Arabic, Gestational diabetes mellitus, Self-care, Self-management

## Abstract

**Background and purpose:**

There is a lack of validated tools to assess adherence to gestational diabetes (GDM) management plan among women with GDM. This study aimed to translate the Summary of Diabetes Self-Care Activity (SDSCA) into Arabic, culturally adapt it, and test its psychometric properties among women with GDM.

**Methods:**

A multiphase study was used to translate and evaluate the psychometric properties of the Arabic version of SDSCA using the following steps: (1) cultural and linguistic validation; (2) content and face validity testing; (3) construct validity testing; and (4) internal validity testing. Nineghty pregnant women with GDM were recruited to meet the purpose of this study.

**Results:**

The Arabic version of the adapted SDSCA tool revealed adequate content validity, satisfactory internal consistency (Cronbach’s alpha = 0.74), and test-retest reliability (Pearson correlation coefficient = .67). Exploratory factor analysis revealed three factors that fit data satisfactory: diet, exercise, and blood glucose monitoring.

**Conclusions:**

This study showed that the adapted Arabic SDSCA tool is an easy, valid, and reliable tool to assess pregnant women’s adherence to GDM management plan.

**Supplementary Information:**

The online version contains supplementary material available at 10.1186/s12884-022-04897-4.

## Introduction

Diabetes Mellitus (DM) remains a global threat to health. Gestational diabetes mellitus (GDM) is a type of DM that creates an additional burden to the healthcare system. GDM is a significant prenatal metabolic condition of pregnancy defined as “diabetes diagnosed during the second and third trimester of pregnancy with no previous history of DM” ([37], p25). Worldwide, hyperglycemia in pregnancy is increasing dramatically, and the global prevalence was estimated to be 16.2%, with the highest prevalence was in South East Asia (24.2%) followed by the Middle East and North Africa (21.8%) [16, 18]. In 2017, it was estimated that 86% of live births with hyperglycemia were due to GDM [18]. In the Middle East, the prevalence of GDM ranges between 5 and 37% [3, 6]. Compared to European and other Western countries, GDM prevalence in the Middle East is higher [3, 6, 16]. In Oman, the reported prevalence of GDM in 2017 was 15% [33], and the incidence of GDM as reported in 2021 was 48.5%, with 74% of the GDM women were managed by diet, and the remaining were treated with hypoglycemic agents [5].

Like DM, improper management of GDM causes various complications for mothers and neonates. At the maternal level, GDM was linked to increased risk for cesarean deliveries, gestational hypertension, and development of type 2 diabetes (T2DM) and cardiovascular diseases after pregnancy [8, 9, 18, 30, 39, 40]. A study in Lebanon reported that the risk to develop T2DM in GDM is 4-times higher than for non-GDM mothers [17]. Further, mothers with GDM are at a higher risk of delivering premature and low birth infants in subsequent pregnancies [40]. At the neonatal level, GDM causes hypoglycemia, hyperbilirubinemia, respiratory distress, shoulder dystocia, brachial plexus injury, and macrosomia [8–10, 18, 27, 39, 40]. In addition, children of GDM mothers are at a higher risk of developing obesity, T2DM, hyperlipidemia, and cardiovascular and congenital heart diseases [8, 9, 18, 27, 40]. Besides, a systematic review and meta-analysis of 24 studies have shown that neonatal respiratory distress syndrome is two times higher with GDM [25]. The economic burden of GDM has increased drastically due to the increment in the number of hospitalizations, clinical visits and exams, complications of GDM, and the use of hypoglycemic and insulin agents [10, 39]. An Italian study reported that women with GDM cost 29.2% more than non-GDM [10]. Hospitalized GDM mothers and their neonates cost 15–49% higher than non-GDM mothers and neonates [10, 24].

Due to the increased prevalence, complications, and economic burdens on mothers, offspring, and the healthcare system, management of GDM is necessary [8]. Overweight, obese, sedentary, and unhealthy lifestyles are leading factors to the development of GDM [8, 10]. For instance, excessive weight gain during pregnancy in mothers with GDM was significantly linked to cesarean deliveries, gestational hypertension, hypoglycemic agents use after delivery, and increased infant birth weight [9, 29, 40, 43]. Further, excessive weight gain between pregnancies was connected with a higher risk of GDM in the subsequent pregnancy [28, 40]. A systematic review and a meta-analysis of 13 studies have reported that inter-pregnancy weight loss decreases the risk of developing GDM in subsequent pregnancies [28]. Furthermore, adherence to lifestyle modifications and self-care management (e.g., weight, diet, and exercise) showed their effectiveness in achieving glycemic control and reducing complications in GDM [8]. Thus, commitment to self-care behaviors, including lifestyle interventions and medication, is the cornerstone of GDM management and control [11].

In DM, adherence to self-care behaviors determines management effectiveness and glycemic control [21]. Strategies empowering self-care behavior among mothers with GDM led to increased adherence to diet, drug use, physical activity, and self-monitoring of blood glucose; increased glycemic control [22, 23, 44]; reduced rates of cesarean delivery and macrosomia [31]; reduced rate of newborn hospitalization [23]; and reduced anxiety [22]. Moreover, mothers with GDM reported the need for support from healthcare providers and families to engage in and adhere to self-care activities [1, 12].

Although adherence to self-care behaviors has been associated with improved glycemic control among patients with T2DM, there is a scarcity of evidence that examined the impact of adherence to self-care behaviors among pregnant women with GDM. This is maybe because there is no scale specifically designed to assess adherence to self-care behaviors among pregnant women with GDM. Adherence to self-care behaviors in DM and GDM entails multiple dimensions and, thus, requires assessment and evaluation to achieve the best outcomes. Therefore, to achieve this, healthcare providers need to utilize a valid, reliable, and culturally appropriate instrument to assess adherence to self-care behaviors. Many tools available to measure adherence to self-care behaviors among patients with DM, one of which is widely utilized is the Summary of Diabetes Self Care Activities (SDSCA) [38]. The reliability and the validity of the tool have been well established [38].

To our knowledge and based on the reviewed literature, there is no Arabic version of SDSCA used among pregnant women with GDM. Therefore, this study aimed to translate, culturally adapt and assess the psychometric properties of the original version of SDSCA among pregnant women with GDM, the Arabic version of SDSCA. The current study is the first study to adapt SDSCA among a sample of pregnant women with GDM. The utilization of the adapted SDSCA tool would enable researchers in the field of GDM to conduct future studies in pregnant women with GDM and help healthcare providers to assess women with GDM adherence to the gestational diabetes management plan. Precisely, it will guide developing interventions to improve pregnant women’s adherence to the GDM management plan, which will subsequently support this population in managing their GDM and improving their pregnancy outcomes.

## Methods

### Design and setting

We conducted this psychometric study between July and November 2017 at antenatal clinic at a major governmental hospital located in the Sultanate of Oman. This hospital provides healthcare services to patients from various places across the Sultanate of Oman.

### Instrument

Summary of Diabetes Self-Care Activity (SDSCA) is a valid and reliable tool designed to measure the adherence to self-care behaviors undertaken by T2DM patients over the past 7 days [38]. This instrument was developed and updated based on seven previous studies [38]. The original tool consists of 11 items evaluating five domains of diabetes-specific management, including diet (4 items), exercise (2 items), glucose monitoring (2 items), foot care (2 items), and smoking (1 item). Response categories in SDSCA range from 0 to 7 days a week. Using the average mean of items, the total score in this tool ranged from 0 to 35, with a higher score suggesting higher adherence to healthy behaviors. The internal reliability of the original tool has been well established with high inter-item correlation (mean = 0.47) and moderate test-retest correlation (mean = 0.40) [38]. The approval to use the tool was obtained from the developer of the tool.

### Procedure

Institutional ethical approval was obtained before the initiation of the study. Written informed consent was obtained from all study participants. The World Health Organization’s steps of translation and adaptation of instruments were used to guide the translation and validation of the adapted SDSCA tool [42]. The authors followed four steps to examine the instrument’s validity and reliability: forward translation, testing instrument validity (namely, the face, content, and construct validity), backward translation and pilot testing.

The first author, a nursing faculty specialized in GDM, and coauthors reviewed the original SDSCA and amended the subscales accordingly in the initial phase, considering the nature of the GDM management plan and the Arabic culture. Subscales of foot care (item # 9 & 10) and smoking (item # 11) that do not apply to the GDM management plan were excluded from the tool. The remaining subscales (i.e., diet, exercise, and glucose monitoring subscales) were the scope of the current study translation and validation (Table 1).

**Table 1:** Original SDSCA tool with the list of items removed

No.	Original SDSCA Tool	Action	Reason
	**Diet**		
1.	How many of the last 7 days have you followed a healthful eating plan?	Maintained	Part of GDM management plan
2.	On average, over the past month, how many days per week have you followed your eating plan?		
3.	On how many of the last 7 days did you eat five or more servings of fruit and vegetables?		
4.	On how many of the last 7 days did you eat high fat foods such as red meat or full-fat dairy products?		
	**Exercise**		
5.	On how many of the last 7 days did you participate in at least 30 minutes of physical activity? (Total minutes of continuous activity, including walking)	Maintained	Part of GDM management plan
6.	On how many of the last 7 days did you participate in a specific exercise session (such as walking, biking) other than what you do around the house or as part of your work?		
	**Blood Sugar Testing**		
7.	On how many of the last 7 days did you test your blood sugar?	Maintained	Part of GDM management plan
8.	On how many of the last 7 days did you test your blood sugar the number of times recommended by your health care provider?		
	**Foot Care**		
9.	On how many of the last 7 days did you check your feet?	Removed	Not applicable for women with GDM
10.	On how many of the last 7 days did you inspect the inside of your shoes?		
	**Smoking**		
11.	Have you smoked a cigarette – even one puff – during the past 7 days?	Removed	Not applicable for women with GDM

### Translation and validation process

#### Forward translation

The first step included translation of the modified SDSCA scale from English into Arabic by two professional bilingual translators.

#### Testing instrument validity

Two Arabic researchers compared and analyzed the two translated versions until they concluded that the Arabic version best reflects the language and concepts of the English version. As a result, the Arabic version’s first draft was completed. The translated Arabic version of the adapted SDSCA was then presented to a panel of five experts knowledgeable about Arabic culture in the second phase, who assessed the content validity of the Arabic version of the adapted SDSCA. Two diabetes nurses, one diabetologist, and two nursing lecturers comprised the expert panel. Panel members rated each item on a four-point scale (4 = very relevant, 3 = relevant with some adjustment to phrasing, 2 = only relevant if the phrasing is profoundly adjusted, and 1 = not relevant). The panelists were also asked to remark on the clarity, accuracy, and cultural relevance of the wording for each item. The overall structure of the Arabic version of the adapted SDSCA was modified, and the individual items were corrected (total items = 8) based on the panel’s comments. Item Content Validity Index (I-CVI) and Scale Content Validity Index (S-CVI) were used to establish the content validity of the tool [35]. In the I-CVI, a score of 0.78 or higher shows satisfactory content validity. The CVI processes’ outcomes are reported in the Results section.

#### Backward translation

One experienced multilingual translator reverse-translated the second Arabic version of the adapted SDSCA tool into the English language. The back-translated copy resembled the original SGDSCA tool extremely well.

#### Pilot testing

Five pregnant women with GDM from the Antenatal Clinic were pilot tested with the second version of the adapted Arabic SDSCA. The chosen participants were representative of the targeted population but were not included in data analysis. When they were waiting for their prenatal checkup, the five participants completed the instrument twice (at baseline and after 4 weeks). To reduce the likelihood of recall bias, a retest was given 4 weeks after the initial test. As part of the survey, participants also had an opportunity to comment and suggest how the scale could be improved and how to make each item more transparent and understandable. The scale’s readability was based on the SMOG index for readers in 8th grade.

#### Final version

The final version of the adapted Arabic SDSCA was created based on participants’ input in the pilot testing, and it was validated among 90 Omani pregnant women with GDM. The study followed Jum and Ira [19] recommendation (i.e., respondent-to-item ratio 10:1) to calculate the required sample size for this study. Since the adapted Arabic SDSCA scale has 8 items, a total of 80 participants were required to be included in this validation study. We expected an attrition rate of 10%; therefore, the final approximation for the overall sample was 90 participants. Participants for the study were Omani women, 18 years or older, diagnosed with GDM, attending the study setting during the study period, and speaking, reading and writing in Arabic language. The study excluded women with type 1 or type 2 diabetes and women with mental illness. Participants completed the adapted Arabic SDSCA questionnaier twice at baseline and after 4 weeks while waiting for their prenatal appointment.

### Data analysis

Data were analyzed using Statistical Package for the Social Sciences software (SPSS), version 24 [15]. Cronbach’s alpha was calculated for the overall adapted Arabic SDSCA scale to measure internal consistency reliability. A Cronbach alpha value of greater than .7 is considered as acceptable. The Pearson correlation coefficient was used to examine test-retest reliability with a four-week delay between the two administrations of the scale. The item-content validity index (I-CVI) and scale-content validity index (S-CVI) for items in the adapted Arabic copy of SDSCA were calculated to assess content validity. An acceptable content validity score was more than .78 for the I-CVI and more than .8 for the S-CVI [26]. Construct validity of the tool was examined using exploratory factor analysis (EFA). Kaiser-Meyer-Olkin (KMO) was used to measure data adequacy and Bartlett’s test of sphericity to test for factors with inter-related items.

## Results

### Demographic data

This study involved 90 Omani women with GDM who completed the adapted Arabic SDSCA scale questionnaire. No participants dropped out during the pre- or the post-test periods. Women ranged in age from 19 to 43 years (Mean = 33.5, SD = 5.10) and in the body mass index from 17.4–60.0 kg/m^2^. Most (85.5%) of the women had at least a high school level of education (Table 2).

**Table 2:** Sample demographic characteristics and inter-group comparison

Sample Characteristic	Total Sample *N* = 90
Age (years), m (SD)	33.5 (5.10)
Education Level, n (%)	
Less than High School	13 (14.4)
High School Graduate	30 (33.3)
Some College/ College Graduate	35 (44.4)
Graduate Degree	7 (7.8)
Work Status, n (%)	
Working	49 (54.4)
Not Working	41 (45.6)
Body Mass Index, m (SD)	29.0 (7.02)
Gravida, m (SD)	4.0 (2.38)
Para, m (SD)	2.3 (1.82)
Weeks of Gestation at GDM Diagnosis, m (SD)	20.1 (7.49)

*GDM* Gestational Diabetes Mellitus

### Content validity

For items in the adapted Arabic SDSCA, the estimated content validity index (CVI) was between .8 and 1, suggesting satisfactory validity. As a result of the content validity findings, the adapted Arabic version of SDSCA for pregnant women with GDM was developed.

It included a total of eight items and three subscales, which are the diet subscale (4 items), physical activity subscale (2 items), and the self-monitoring of blood glucose subscale (2 items) (Table 1). Response categories in the adapted Arabic SDSCA range from 0 to 7 days a week. Using the average mean of items, the mean score of the adapted Arabic SDSCA lies between 0 and 7, with a higher mean score suggesting higher engagement in healthy behaviors.

### Construct validity

The EFA results for the adapted Arabic version of the SDSCA were KMO = 0.53, Bartlett’s test of sphericity was chi-square (df = 28) = 171.33, *p*-value 0.001. An orthogonal rotation was utilized because the component correlation matrix’s coefficients were less than .32. Using the parallel analysis engine and the eigenvalue technique, three factors (eigenvalue > 1) were extracted. The extraction sum of squares loadings explained 64.67% of the overall variance. Table 3 shows the factor loadings for the three-factor solution. Cronbach’s alphas for factors range from .58–.88.

**Table 3:** Final set of underlying factors identified by exploratory factor analysis

Description of Items	Factor			Communal
	1	2	3	
**Diet**				
How many of the last 7 days have you followed a healthful eating plan?	.88			.77
On average, over the past month, how many days per week have you followed your eating plan?	.79			.67
On how many of the last 7 days did you eat five or more servings of fruits and vegetables?	.41			.22
On how many of the last 7 days did you eat high-fat foods, such as red meat or full-fat dairy products?	.40			.50
**Exercise**				
On how many of the last 7 days did you participate in at least 30 minutes of physical activity?			.74	.62
On how many of the last 7 days did you participate in a specific exercise session (such as such swimming, walking, biking) other than what you do around the house or as part of your work?			.86	.76
**Blood Glucose Monitoring**				
On how many of the last 7 days did you test your blood sugar?		.88		.85
On how many of the last 7 days did you test your blood sugar the number of times recommended by your health care provider?		.86		.79
**Cronbach’s Alpha**	**.58**	**.84**	**.88**	

### Internal consistency reliability

The adapted Arabic SDSCA had a Cronbach’s alpha coefficient of .74, indicating satisfactory (standardized) internal consistency. Correlation coefficients between items varied from .12 to .58. When one item was deleted at a time and Cronbach’s alpha was recalculated, the coefficients ranged from .69 to.77 (Table 3). The Pearson correlation coefficient was used to examine test-retest reliability. With a Pearson correlation coefficient of .67, the adapted Arabic SDSCA instrument demonstrated a strong significant association with time, as shown in Table 4. Overall, the internal consistency and test-retest results (Table 5) showed that the study instrument was reliable and consistent throughout time.

**Table 4:** Total to item correlation among items in tool (overall Cronbach’s alpha = .74)

Item	Corrected Item-Total Correlation	Cronbach’s Alpha if item Deleted
1. How many of the last 7 days have you followed a healthful eating plan?	.53	.70
2. On average, over the past month, how many DAYS PER WEEK have you followed your eating plan?	.58	.69
3. On how many of the last 7 days did you eat five or more servings of fruits and vegetables?	.34	.73
4. On how many of the last 7 days did you eat high-fat foods, such as red meat or full-fat dairy products?	.12	.77
5. On how many of the last 7 days did you participate in at least 30 minutes of physical activity?	.55	.69
6. On how many of the last 7 days did you participate in a specific exercise session (such as such swimming, walking, biking) other than what you do around the house or as part of your work?	.49	.71
7. On how many of the last 7 days did you test your blood sugar?	.52	.70
8. On how many of the last 7 days did you test your blood sugar the number of times recommended by your health care provider?	.42	.72

**Table 5:** Test-retest reliability

Instrument	Mean	SD	Pearson correlation coefficient	***P*** value
Summary of Diabetes Self-Care Activities (SDSCA)			0.57	<.001
SDSCA Score, Test 1	3.14	1.07		
SDSCA Score, Test 2	4.10	1.24		
Diet, Test 1	4.79	1.21	0.55	<.001
Diet, Test 2	4.00	1.28		
Pysical Activity, Test 1	2.1	2.05	0.51	<.001
Pysical Activity, Test 2	3.75	2.41		
Blood Glucose Monitoring, Test 1	2.48	2.04	0.29	<.001
Blood Glucose Monitoring, Test 2	3.08	1.85		

## Discussion

Lack of management for GDM can negatively influence women, offspring, and the healthcare system [8]. Adherence to self-care behaviors is essential in managing GDM [21]. There is no tool available to assess adherence to self-care behaviors among pregnant women with GDM in Arabic context. However, several valid and reliable tools are available to evaluate adherence to self-care behaviors designed for individuals with T2DM, one of which is the SDSCA. The self-care behaviors of GDM are almost comparable to the self-care behaviors of T2DM, and many items from the original SDSCA would apply to pregnant women with GDM [41].

A valid, reliable, and culturally appropriate instrument is needed to evaluate and assure adherence to self-care behaviors among pregnant women with GDM. Therefore, this study translated the original SDSCA into Arabic and evaluated its psychometric properties among pregnant women with GDM. The results indicated that the culturally adapted Arabic-SDSCA is acceptable. An instrument’s translation and back-translation necessitate literal translation and cultural adaptation and application to the target group. In the current study, two out of the five subscales in the original SDSCA tool were not applicable and valid to the GDM-management plan. The content validity index (CVI) of the relevant items was between .8 and 1 for items in the adapted SDSCA [i.e., diet subscale (4 items), physical activity subscale (2 items), and the self-monitoring of blood glucose subscale (2 items)], indicating acceptable content validity. Therefore, the final results of the content validity support the adapted SDSCA Arabic version for pregnant women with GDM. For this study, KMO was greater than 0.50, indicating that the sample size was adequate in performing EFA, and items can be grouped into underlying factors [14]. Bartlett’s test of sphericity was significant, indicating that there were factors with inter-related items.

There is no other study that we are aware of that has looked into the validity or reliability of the original SDSCA instrument in pregnant women with GDM. Apart from T2DM patients, the original SDSCA was tested in Pakistan among middle-aged patients using the Urdu version, with a CVI score of .92 for the ten relevant items (out of 14), showing good content validity. Based on data collected from 30 middle-aged participants, the items’ internal consistency was acceptable (0.79) [4]. This also enables the original SDSCA to be used for populations other than patients with T2DM, such as patients and pregnant women with GDM. The feasibility of the original SDSCA for different populations, for example, patients with type 1 DM or children with obesity, may be explored in future research.

The EFA in the current study confirmed that the adapted Arabic SDSCA tool contains three factors (i.e., diet, exercise, and blood glucose testing). These results are slightly different than the previous studies tested among patients with type 2 diabetes which revealed four factors {i.e., diet, exercise, blood glucose testing, and foot care} [2, 7, 20, 32]. This minor difference in the subscales found between the aforementioned versions and the current study may be associated with the temporary nature of the GDM. The foot care is not typically included in the GDM management plan and may not apply to pregnant women with GDM. However, the eight items solutions in the current study showed similar distribution within the three-factor model of previous studies [20, 32]. This equal distribution also may indicate that items included in the three subscales diet, exercise, and glucose monitoring are universal and not be impacted by cultural issues.

The original SDSCA was also translated and evaluated, and it was found to be valid and reliable among type 2 diabetes patients from Indonesia, Ghana, Germany, and Malaysia [7, 20, 32, 36]. An Arabic version of the original SDSCA was tested among 243 patients with T2DM, where test-retest reliability was .91 and Cronbach’s alpha coefficient of the total scale was 0.76. An Arabic version of the original SDSCA has never been tested for its reliability among pregnant women with GDM.

Our study showed test-retest reliability of .67, and Cronbach’s alpha of .74, indicating a good internal consistency and homogeneity of scale items. Due to the low number of items in some scales and its impact on Cronbach’s alpha, the inter-item correlation was chosen to estimate the reliability of this instrument [34]. Low to moderate item-to-item correlation indicates no redundancy of items in each subscale. Test-retest reliability showed excellent stability over time. Test-retest reliability value greater than .75 is considered excellent reliability [13]. The internal consistency and test-retest results demonstrated that the adapted SDSCA tool is a reliable and consistent instrument over time.

The current study’s key strength is having an adequate sample size and the use of established research processes and psychometric evaluation procedures. However, this study had certain drawbacks, including the lack of criterion validity. We could not calculate the correlation between the adapted SDSCA scores and Hemoglobin A1C (HbA1c) scores. Data on blood sugar control (HbA1c) was unavailable for all study participants. Furthermore, because the study subjects were recruited from a single Arabic country, the findings’ generalizability would be limited. As a result, continued testing of the instrument among Arabic pregnant women with GDM from multiple sites across the Middle East region is required.

## Conclusion

The goal of this study was to translate the original SDSCA, culturally adapt it, and examine its psychometric qualities among pregnant women with GDM. Overall, the adapted SDSCA has adequate psychometric qualities when used among Arabic pregnant women with GDM, according to the findings of this study. This tool can be used in research and practice to assess women with GDM’s adherence to their GDM management plan.

## Supplementary Information

**Additional file 1.** The Arabic translation of the Summary of Diabetes Self-Care Activities (SDSCA).

## Data Availability

The datasets generated and/or analysed during the current study are not publicly available due [restrictions by the Research and Ethics Committee in the College of Medicine at Sultan Qaboos University to protect the participants’ privacy] but are available from the Principal Investigator (Iman Al Hashmi) on reasonable request.

## Abbreviations

DM: Diabetes mellitus
GDM: Gestational diabetes mellitus
HbA1c: Hemoglobin A1C
EFA: Exploratory factor analysis
I-CVI: Item-content validity index
KMO: Kaiser-Meyer-Olkin
S-CVI: Scale-content validity index
SDSCA: Summary of Diabetes Self-Care Activity
T2DM: Type 2 diabetes mellitus
