# Supplementary material for: Translation and psychometric validation of the Arabic version of Summary of the Diabetes Self-Care Activities (SDSCA) among pregnant women with gestational diabetes
Source: BMC Pregnancy Childbirth. 2022 Jul 14;22:563. doi: 10.1186/s12884-022-04897-4 (PMC9284767; doi:10.1186/s12884-022-04897-4)
Supplement: Supplementary file 1 — Additional file 1. The Arabic translation of the Summary of Diabetes Self-Care Activities (SDSCA). [file 12884_2022_4897_MOESM1_ESM.docx]

The Summary of Diabetes Self- Care Activities

The questions below ask you about your diabetes self-care activities during the past 7 days. If you were sick during the past 7 days, please think back to the last 7 days that you were not sick.

| **Diet** | Number of days | | | | | | | |
| --- | --- | --- | --- | --- | --- | --- | --- | --- |
| 1. How many of the last SEVEN DAYS have you followed a healthful eating plan? | 0 | 1 | 2 | 3 | 4 | 5 | 6 | 7 |
| 2. On average, over the past month, how many DAYS PER WEEK have you followed your eating plan? | 0 | 1 | 2 | 3 | 4 | 5 | 6 | 7 |
| 3. On how many of the last SEVEN DAYS did you eat five or more servings of fruits and vegetables? | 0 | 1 | 2 | 3 | 4 | 5 | 6 | 7 |
| 4. On how many of the last SEVEN DAYS did you eat high fat foods such as red meat or full-fat dairy products? | 0 | 1 | 2 | 3 | 4 | 5 | 6 | 7 |

| **Exercise** | Number of days | | | | | | | |
| --- | --- | --- | --- | --- | --- | --- | --- | --- |
| 1. On how many of the last SEVEN DAYS did you participate in at least 30 minutes of physical activity? | 0 | 1 | 2 | 3 | 4 | 5 | 6 | 7 |
| 2. On how many of the last SEVEN DAYS did you participate in a specific exercise session (such as swimming, walking, biking) other than what you do around the house or as part of your work? | 0 | 1 | 2 | 3 | 4 | 5 | 6 | 7 |

| **Blood Sugar Testing** | Number of days | | | | | | | |
| --- | --- | --- | --- | --- | --- | --- | --- | --- |
| 1. On how many of the last SEVEN DAYS did you test your blood sugar? | 0 | 1 | 2 | 3 | 4 | 5 | 6 | 7 |
| 2. On how many of the last SEVEN DAYS did you test your blood sugar the number of times recommended by your health care provider? | 0 | 1 | 2 | 3 | 4 | 5 | 6 | 7 |

ملخص نشاطات العناية الذاتية بمرض سكر الحمل، (المتعلقة بالتغذية والتمارين الرياضيه وفحص سكر الدم)

الأسئله التاليه تدور حول الأنشطة المتعلقة بعنايتك بمرض سكري الحمل خلال الأيام السبعة الماضية. إذا كنتي مريضه خلال هذه الأيام ، نرجو منك أن تتذكري الأيام السبعة الأخيرة قبل أن تمرضي.

| **التغذية** | عدد ألايام | | | | | | | |
| --- | --- | --- | --- | --- | --- | --- | --- | --- |
| ١. خلال السبعة أيام الماضية، كم عدد الأيام التي إتبعت فيها نظام غذائي صحي؟ | 0 | 1 | 2 | 3 | 4 | 5 | 6 | 7 |
| ٢. خلال الشهر الماضي، بمعدل كم يوم في الأسبوع اتبعت خطتك للأكل؟ | 0 | 1 | 2 | 3 | 4 | 5 | 6 | 7 |
| ٣. خلال السبعة أيام الماضية، كم عدد الأيام التي أكلت فيها خمسة حصص أو أكثر من الفاكهة والخضروات؟ | 0 | 1 | 2 | 3 | 4 | 5 | 6 | 7 |
| ٤. خلال السبعه أيام الماضيه، كم عدد الأيام التي أكلت فيه أطعمة غنية بالدهون كاللحم الأحمر ومشتقات الحليب الكاملة الدسم؟ | 0 | 1 | 2 | 3 | 4 | 5 | 6 | 7 |

| **التمارين الرياضية** | عدد ألايام | | | | | | | |
| --- | --- | --- | --- | --- | --- | --- | --- | --- |
| ١. خلال السبعة أيام الماضية، كم عدد الأيام التي مارست فيها أنشطه رياضيه لمده لا تقل عن ٣٠ دقيقه؟ | 0 | 1 | 2 | 3 | 4 | 5 | 6 | 7 |
| ٣. خلال السبعة أيام الماضية، كم عدد الأيام التي مارست فيها نشاط رياضي معين مثل (السباحة، المشي)، عدا الذي تقوم به حول المنزل أو كجزء من عملك؟ | 0 | 1 | 2 | 3 | 4 | 5 | 6 | 7 |

| **فحص سكر الدم** | عدد ألايام | | | | | | | |
| --- | --- | --- | --- | --- | --- | --- | --- | --- |
| ١. خلال السبعة أيام الماضية، كم عدد الأيام التي فحصت فيها سكر الدم؟ | 0 | 1 | 2 | 3 | 4 | 5 | 6 | 7 |
| ٣. خلال السبعة أيام الماضية، كم عدد الأيام التي فحصت فيها سكر الدم حسب العدد المذكور في تعليمات طبيبك؟ | 0 | 1 | 2 | 3 | 4 | 5 | 6 | 7 |
